# Molecular Characterization and Functional Insights into Goose IGF2BP2 During Skeletal Muscle Development

**DOI:** 10.3390/ani16010058

**Published:** 2025-12-24

**Authors:** Cui Wang, Yi Liu, Jiuli Dai, Shufang Chen, Daqian He

**Affiliations:** 1 Institute of Animal Science and Veterinary Medicine, Shanghai Academy of Agricultural Sciences, Shanghai 201106, China; cuiwang518@saas.sh.cn (C.W.); liuyi20031194@163.com (Y.L.); 2 Ningbo Academy of Agricultural Sciences, Ningbo 315040, China; 13858357201@163.com (J.D.); 13606780161@163.com (S.C.)

**Keywords:** goose, IGF2BP2, expression profile, SMSCs, overexpression, RNA-seq

## Abstract

Insulin-like growth factor 2 mRNA-binding protein 2 (IGF2BP2) is an RNA-binding protein known to play critical roles in metabolism, cell proliferation, and tumorigenesis. Although its involvement in muscle development has been documented in several species, the function of goose IGF2BP2 remains largely unexplored. In this study, we cloned and characterized the full-length cDNA and genomic DNA sequences of goose IGF2BP2. The cDNA is 2957 bp in length and contains a 1662 bp open reading frame encoding a 553-amino acid protein with five conserved RNA-binding domains. The genomic sequence spans 12,183 bp and consists of 12 exons and 11 introns. A total of 60 genetic variants were identified, including a deletion of a G base at position 2299 (g.2299delG) that results in a frameshift mutation. Expression analysis revealed high levels of IGF2BP2 mRNA in the liver, heart, and muscle tissues of female geese across embryonic (E25d), growing (A70d), and laying (L270d) stages, consistent with a potential role in muscle development (*p* < 0.05). Functionally, overexpression of IGF2BP2 in skeletal muscle satellite cells (SMSCs) was associated with significant changes in the expression of several genes linked to muscle development and signaling pathways, including upregulation of IGF1, EGFR, FGF19, BMP6, BMP2, ACVR1C and WNT5A and downregulation of MYBPC3, NODAL, HOXD13, TNXB, and ADD2 (*Padj* < 0.01). Furthermore, protein–protein interaction (PPI) network analysis of these genes suggests that IGF2BP2 may coordinate key genes, contributing to its potential role in skeletal muscle development in geese.

## Simple Summary

This study investigates the role of insulin-like growth factor 2 mRNA-binding protein 2 (IGF2BP2) in geese. We found that IGF2BP2 is highly expressed in muscle tissues and influences key cellular processes. By increasing its activity in muscle cells, we demonstrated that IGF2BP2 may regulate a network of genes critical for muscle development. Our findings indicate that IGF2BP2 acts as an important regulator of muscle development in geese.

## 1. Introduction

Insulin-like growth factor 2 mRNA-binding proteins (IGF2BPs or IMPs) constitute a conserved family of RNA-binding proteins with three known members: IGF2BP1, IGF2BP2 and IGF2BP3. These proteins play pivotal roles in post-transcriptional gene regulation, critically influencing cellular processes such as proliferation, differentiation, migration, and metabolism [1,2,3]. As a core member of this family, IGF2BP2 functions as an N6-methyladenosine (m6A) reader, modulating the stability, localization, and translation of target mRNAs involved in metabolic and developmental pathways [4].

The role of IGF2BP2 in myogenesis is increasingly recognized across species. In mice, IGF2BP2 promotes myoblast proliferation by binding to and stabilizing key transcripts such as Myc and IGF1R [5,6]. Its transcription during muscle development is also modulated by factors including HMGA2 and HMGB2 [7,8]. Beyond murine models, genome-wide selective sweep analysis in goats has identified IGF2BP2 as a candidate gene affecting litter size, potentially through functions in germ cell and embryonic development [9]. In chickens, IGF2BP2 has been associated with skeletal muscle growth and testicular traits [10,11]. In ducks, genomic selective sweeps analysis revealed that IGF2BP2 is linked to muscle development in the Huitang breed [12]. Similarly, a relationship between IGF2BP2 gene polymorphism and growth and reproduction traits has been reported in pigeons [13]. Collectively, these findings underscore the conserved and multifaceted role of IGF2BP2 in vertebrate growth and reproduction.

Despite these insights, a significant knowledge gap remains regarding IGF2BP2 in goose (*Anser cygnoides*), an economically vital poultry species prized for its meat. In geese, skeletal muscle development is the primary determinant of meat yield and quality—traits of major agricultural significance. However, the expression patterns, molecular characteristics, and functional roles of IGF2BP2 in goose skeletal muscle formation remain largely unexplored. Understanding its function in geese is essential not only to complete the comparative biological framework across species but also for its potential relevance to poultry production. Elucidating how IGF2BP2 governs myogenesis may reveal novel molecular targets for genetic selection or management strategies aimed at improving muscle growth efficiency and meat yield in geese.

Therefore, this study aimed to address these gaps by hypothesizing that goose IGF2BP2 is a key regulator of skeletal muscle development. Our specific objectives were to (1) clone and characterize the goose IGF2BP2 gene, (2) analyze its expression patterns across various tissues and at different developmental stages, and (3) investigate its functional role in the proliferation and differentiation of goose skeletal muscle satellite cells (SMSCs). Our findings provide novel insights into the molecular mechanisms underlying muscle development in geese and may inform future breeding strategies aimed at enhancing meat production.

## 2. Materials and Methods

### 2.1. Animals and Sample Collection

Goose tissue samples were obtained across three developmental stages: embryos at embryonic day 16 (E16d) and day 25 (E25d; *n* = 20 per stage), adult female at 70 days (A70d; *n* = 4), and laying geese at 270 days (L270d; *n* = 4). All geese were supplied by the Wenjie Goose Breeding Department of Xiangshan Co., Ltd. (Ningbo, China). Embryos were incubated in a standard commercial incubator (Zhonglian, Shanghai, China), and post-hatch geese were raised under standard commercial management with access to open ground and a swimming pool.

Sex determination for E16d and E25d embryos was performed via PCR using CHD1 gene specific primers [14] (Table 1). Leg muscle tissue from A70d geese was specifically used for cloning the gIGF2BP2 cDNA sequence. For expression profiling, multiple tissues—including heart, liver, spleen, lung, kidney, breast muscle, leg muscle, brain, skin, muscular stomach, hypothalamus, pituitary, and ovary—were collected at three main stages (E25d, A70d, and L270d). All samples were rapidly frozen in liquid nitrogen and stored at −80 °C until RNA extraction.

All animal experiments were approved by the Institutional Animal Care and Use Committee of Shanghai Academy of Agricultural Sciences (License number: SAAS-SL-2023021).

### 2.2. RNA Extraction, DNA Isolation, and cDNA Synthesis

Total RNA was extracted from tissues samples using Trizol Reagent (Invitrogen, Waltham, MA, USA). Following extraction, the RNA was treated with RNase-free DNaseI (TaKaRa, Dalian, China) to remove genomic DNA. First-strand cDNA was synthesized from the purified RNA with the PrimeScript^TM^ RT Reagent Kit featuring gDNA Eraser (TaKaRa, Dalian, China). The resulting cDNA was diluted to a working concentration of 100–300 ng/µL and storage at −20 °C for subsequent quantitative real-time PCR (qPCR) analysis.

Genomic DNA was isolated from blood samples using the AxyPrep^TM^ Blood Genomic DNA Miniprep Kit (Axygen, Union City, CA, USA). The concentration and purity of all nucleic acid samples (DNA and RNA) were subsequently measured with a Nano-Drop ND-1000 spectrophotometer (Thermo Fisher Scientific, Waltham, MA, USA). Prior to storage at −20 °C, the genomic DNA was adjusted to a final concentration of 200–300 ng/µL for future use.

### 2.3. Molecular Cloning and Sequence Analysis of Goose IGF2BP2 Gene

To clone the goose IGF2BP2 cDNA, initial partial coding sequence (CDS) fragments were amplified using two primer pairs (BP2-F1/R1 and BP2-F2/R2; Table 1). These primers were designed based on conserved regions within the chicken (XM_040679797) and duck (XM_038183827) IGF2BP2 gene sequences. PCR was conducted under the following conditions: initial denaturation at 95 °C for 5 min; 37 cycles of denaturation at 95 °C for 35 s, annealing at a primer-specific temperature for 35 s, and extension at 72 °C for 90 s; followed by a final extension at 72 °C for 5 min. The resulting PCR products were purified, cloned into the pMD19-T vector (TaKaRa, Dalian, China), and commercially sequenced (Tsingke, Beijing, China).

To obtain the full-length cDNA, goose-specific primers were designed from the partial sequences for rapid amplification of cDNA ends (RACE). For 5′-RACE, 10 µg of ovarian total RNA was reverse-transcribed using the SMART RACE cDNA Amplification Kit (Clontech, San Jose, CA, USA). The PCR protocol consisted of 94 °C for 4 min; 35 cycles of 94 °C for 35 s, annealing for 35 s, and extension at 72 °C for 30 s to 2 min (depending on product size); with a final extension at 72 °C for 5 min. The 5′-RACE products were gel-purified, cloned into the pEASY-T1 vector (TransGen, Beijing, China), and sequenced.

The obtained cDNA sequences were assembled using DNAMAN 6.0 software. The open reading frame (ORF) and the deduced amino acid sequence were predicted using SeqMan (version 17.4.1, DNASTAR, Madison, WI, USA). Sequence homology was assessed via NCBI BLAST+ 2.17.0, and multiple sequence alignments were performed using CLUSTALW and visualized with ESPript 3.0.

To isolate the genomic DNA sequence of goose IGF2BP2, 11 overlapping primer pairs (BP2-gF1/gR1 to BP2-gF11/gR11; Table 1) were designed based on the duck IGF2BP2 genomic reference sequence (NW_025927757). These primers were used to amplify overlapping fragments from ZW geese genomic DNA. The purified PCR products were cloned into the pMD19-T vector and sequenced. The final genomic sequence was assembled using SeqMan (DNASTAR).

### 2.4. Identification of Genetic Variants

A DNA pool was constructed by combining genomic DNA extracted from blood samples of 24 ZW geese. The same 11 primer pairs used for genomic cloning (Table 1) were employed to amplify the corresponding regions for variant screening within the ZW goose population. The purified PCR products were sequenced by Sangon Biotech (Shanghai, China). Sequence chromatograms were aligned using SeqMan (DNASTAR), and genetic variants were identified based on consistent nucleotide differences among the sequences.

### 2.5. Tissue-Specific Expression Profiling of Goose IGF2BP2 mRNA

The mRNA expression levels of goose IGF2BP2 across various tissues were quantified using qPCR. Each 20 uL reaction consisted of 10 uL of 2× TB Green Premix Ex Taq II (Tli RNaseH Plus) (Bio-Rad, Hercules, CA, USA), 2 µL of cDNA template, 0.5 µL of each primer (10 µM), and 7 µL of nuclease-free water. Amplification was performed on a Bio-Rad C1000 Touch^TM^ Thermal Cycler (Bio-Rad, Hercules, CA, USA) under the following conditions: initial denaturation at 95 °C for 2 min, followed by 40 cycles of 95 °C for 5 s and 60 °C for 30 s. Gene-specific primers for IGF2BP2 (BP2-QF/QR) and the reference gene β-actin (β-actin-F/R) are listed in Table 1. Amplification specificity was confirmed by melting curve analysis. All reactions were run in triplicate, and relative gene expression was calculated using the 2^−∆∆Ct^ method.

### 2.6. Isolation and Culture of Goose SMSCs

Following sex identification, goose embryos were surface-sterilized with ethanol. Leg muscles were aseptically dissected and thoroughly cleared of visible blood vessels, adipose, and connective tissues. The isolated muscle tissue was finely minced and digested at 37 °C for 50 min in high-glucose DMEM (Corning, Grand Island, NY, USA) containing 2 mg/mL Dispase II (Roche, Basel, Switzerland) and 4 mg/mL Collagenase II (Gibco, Grand Island, NY, USA). The digestion was terminated by adding an equal volume of high-glucose DMEM supplemented with 10% fetal bovine serum (FBS; Lonsera, Ciudad de la Costa, Uruguay, South America). The resulting cell suspension was filtered through a 70 µm cell strainer and centrifuged at 350× *g* for 8 min at room temperature. The pellet was resuspended, and red blood cells were lysed using ACK lysis buffer (Gibco, Grand Island, NY, USA).

The harvested cells were resuspended in growth medium composed of DMEM/F12 (Gibco, Grand Island, NY, USA) supplemented with 10% fetal bovine serum (FBS), 1% penicillin–streptomycin (Gibco, Grand Island, NY, USA), and 5 ng/mL basic fibroblast growth factor (bFGF; R&D Systems, Minnneapolis, MN, USA). Cells were maintained at 37 °C in a humidified 5% CO_2_ incubator (Thermo Fisher Scientific, Waltham, MA, USA). To enrich for SMSCs, a differential adhesion strategy was applied. Briefly, after initial plating for 1 h, the supernatant containing slower-adhering SMSCs was transferred to a fresh culture dish. This step was repeated twice to further reduce contamination by rapidly adhering fibroblasts and other stromal cells. The purity of the isolated SMSCs was assessed by Pax7 Immunofluorescence staining [15]. The enriched SMSCs were then harvested for subsequent functional analyses.

### 2.7. Plasmid Construction, Lentiviral Production, and Cell Transduction

The complete coding sequence of goose IGF2BP2 was amplified and cloned into the lentiviral transfer plasmid pKLV2-U6gRNA5(Empty)-PGKmCherry2AGFP (Addgene, Watertown, MA, USA, #67981) using NotI and EcoRI restriction sites with the ClonExpressII One Step Cloning Kit (Vazyme, Nanjing, China). The resulting overexpression plasmid was designated pKLV2-gIGF2BP2. For the control group, the original empty plasmid (pKLV2-U6gRNA5(Empty)-PGKmCherry2AGFP) was used.

For lentivirus production, 293FT cells cultured in 10 cm dishes at 70–80% confluency were co-transfected using a liposomal transfection reagent (Yeasen, Shanghai, China). For each virus preparation, the transfection mixture contained 5 µg of either the pKLV2-IGF2BP2 transfer plasmid (for overexpression) or the empty control plasmid, along with 4 µg of the psPAX2 packaging plasmid and 2 µg of the pMD2.G envelope plasmid. The DNA was complexed with 27.5 µL of transfection reagent in 500 µL of Opti-MEM. Following a 20 min incubation at room temperature, the DNA–liposome complexes were added to the cells.

Viral supernatant was harvested 72 h post-transfection, filtered through a 0.45 µm membrane, and either used immediately for transduction or stored at −80 °C in aliquots. For transduction, purified goose SMSCs were seeded in 6-well plates. Upon reaching 80–90% confluency, the culture medium cells was replaced with 1 mL of the respective viral supernatant (carrying either the IGF2BP2 overexpression construct or the empty vector) to transduce the cells.

At 72 h post-transduction, cells were harvested. Fluorescence-activated cell sorting (FACS) was performed to isolate the tdTomato-positive cell populations. Cells transduced with the pKLV2-gIGF2BP2 virus were designated as BP2-OE (IGF2BP2-overexpressing), while cells transduced with the empty vector virus served as the control group (BP2-WT). These sorted populations were then subjected to subsequent RNA sequencing analysis.

### 2.8. Library Preparation and Transcriptome Analysis

Total RNA was isolated from positively and negatively sorted cell populations using Trizol Reagent (Invitrogen, Waltham, MA, USA). RNA concentration and purity were quantified with a NanoDrop NC2000 spectrophotometer (Thermo Fisher Scientific, Waltham, MA, USA), and RNA integrity was verified via agarose gel electrophoresis. For each sample, 3 µg of high-quality total RNA fulfilling the quality criteria was subsequently utilized for cDNA library preparation. Libraries were constructed using the NEBNext Ultra II RNA Library Prep Kit for Illumina (New England Biolabs, Ipswich, MA, USA), following the manufacturer’s instructions. Finally, paired-end sequencing was performed on an Illumina NovaSeq 6000 platform (Personal Biotech, Shanghai, China).

Raw paired-end sequencing reads were subjected to quality control and adapter trimming using fastp (v0.22.0), with reads of average quality below Q20 being discarded. High-quality reads were subsequently aligned to the reference genome (Assembly accession: GCF_002166845.1) employing HISAT2 (v2.1.0) [16]. Gene-level read counts were generated with HTSeq (v0.9.1) and normalized as Fragments Per Kilobase of transcript per Million mapped reads (FPKM) [17,18]. Differential expression analysis was conducted using the DESeq (v1.38.3) package, where genes exhibiting an absolute log2FoldChange > 1 and an adjusted *p*-value < 0.05 were defined as differentially expressed genes (DEGs) [19]. The raw RNA-seq data generated in this study have been deposited in the NCBI Sequence Read Archive (SRA) under BioProject accession number PRJNA1369723.

For the functional characterization of DEGs, Gene Ontology (GO) and Kyoto Encyclopedia of Genes and Genomes (KEGG) pathway enrichment analyses were performed. GO enrichment was carried out with the topGO (v2.50.0) R package, while KEGG pathway analysis utilized clusterProfiler (v4.6.0) [20,21]. Significantly enriched terms and pathways were identified based on an adjusted *p*-value threshold of <0.05. Protein–protein interaction (PPI) networks were constructed by mapping DEGs the STRING database [22].

### 2.9. Validation of DEG Results by qRT-PCR

To validate the transcriptome findings, eight key differentially expressed genes (DEGs) involved in skeletal muscle development were selected for qRT-PCR analysis. Gene-specific primers were designed with Oligo 6.0 based on reference sequences from the NCBI database (Table 1). qRT-PCR was performed using the TB Green Premix Ex Taq II (Tli RNaseH Plus) (Bio-Rad, Hercules, CA, USA) on a 384-well C1000 Touch^TM^ Thermal Cycler (Bio-Rad, Hercules, CA, USA). Reaction specificity was confirmed by melting curve analysis.

### 2.10. Statistical Analyses

Gene expression levels were quantified using the 2^−∆∆Ct^ method, with β-actin as the endogenous control. Data are presented as mean ± SEM. Statistical significance was assessed by one-way ANOVA followed by Duncan’s post hoc test using SPSS Statistics 22.0 (SPSS Inc., Chicago, IL, USA). Statistical significance was defined as * *p* < 0.05, ** *p* < 0.01, and *** *p* < 0.001.

## 3. Results

### 3.1. cDNA Sequence Analysis of Goose IGF2BP2 Gene

**Figure 1:**
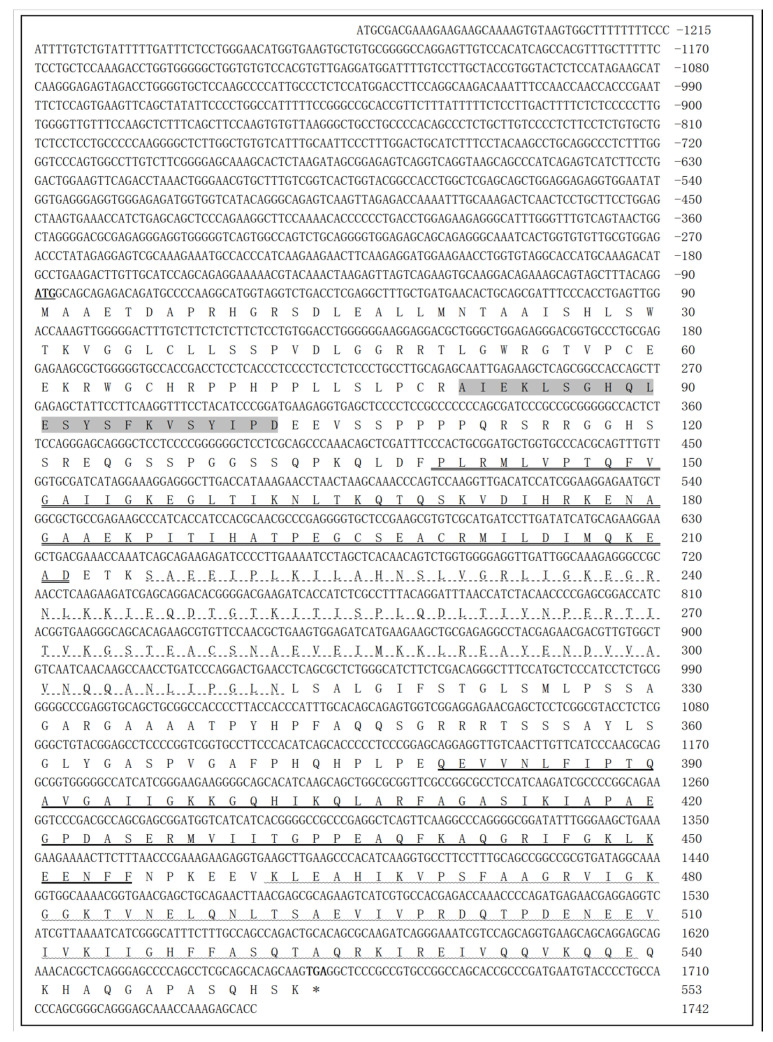
Nucleotide and deduced amino acid sequence of goose IGF2BP2. The ORF spans 1622 bp (position 91–1752) and encodes a 553-amino-acids protein. The start codon (ATG) is bold and underlined; the stop codon (TAA) is indicated by an asterisk (*). The RRM is highlighted in dark gray. The four KH domains are marked as follows: KH1 (double underline), KH2 (dotted underline), KH3 (bold underline) and KH4 (wavy underline).

The full-length coding sequence of the goose IGF2BP2 gene was obtained from a pooled sample of goose tissues using sequence alignment. The cDNA sequence (PP548072) comprises 2957 nucleotides, with an open reading frame (ORF) of 1662 bp, a 5′-untranslated region (UTR) of 1215 bp, and a 3′-UTR of 80 bp (Figure 1). Comparative sequence analysis revealed a high degree of nucleotide identity between this cDNA and IGF2BP2 orthologs from several avian species: *Anas platyrhynchos* (XM_038183827; 96.34%), *Gallus gallus* (XM_015277139; 92.88%), *Falco cherrug* (XM_055723183; 92.59%), and *Coturnix Japonica* (XM_032446565; 92.02%). In contrast, the 5′-UTR showed limited sequence homology with the corresponding regions in other avian species. Bioinformatics analysis indicated that the goose IGF2BP2 gene encodes a 553-amino-acid protein containing five conserved RNA-binding domains: one RNA-recognition motif (RRM) and four K-homology (KH) domains (Figure 1).

**Figure 2:**
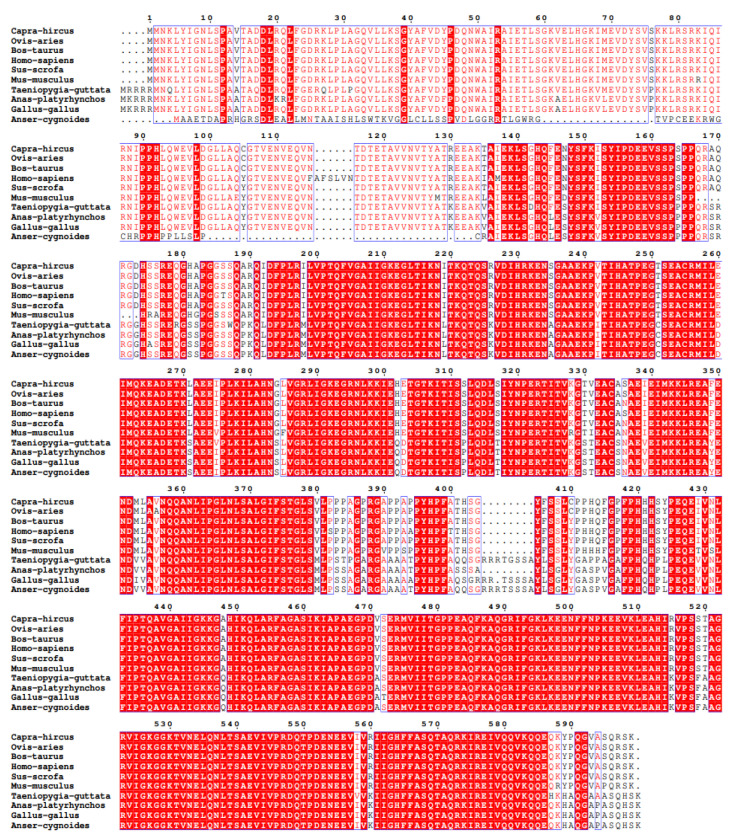
Multiple sequence alignment of IGF2BP2 proteins among selected vertebrates. Strictly identical residues are shown in white characters on a red background; single-letter code with numbering above the alignment. Dashes (–) denote alignment gaps introduced to maximize positional homology; numbers in parentheses at the right margin give the total length of each mature protein.

Multiple sequence alignment revealed that the deduced IGF2BP2 protein in goose is shorter than its orthologs in other avian and mammalian species. Nevertheless, the protein is highly conserved across these species, particularly within the canonical RNA-binding domains (Figure 2).

### 3.2. Genomic Organization and SNP Survey of Goose IGF2BP2

**Figure 3:**
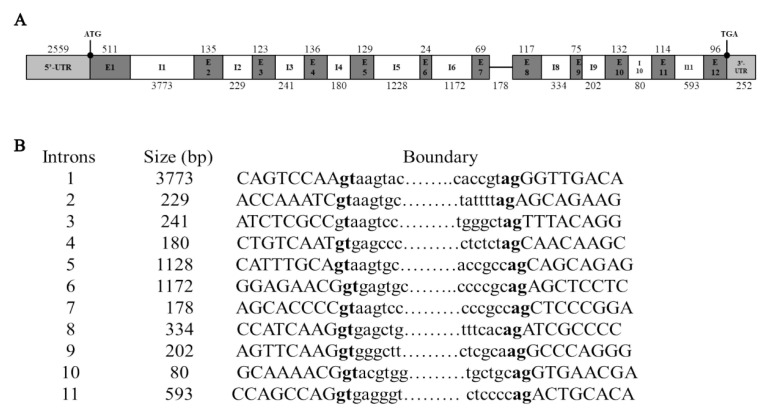
Genomic architecture of the goose IGF2BP2 gene. (**A**) Exon-intron organization. Grey boxes denote exons (E1–E12), connecting lines represent introns. Numbers below boxes indicate exon lengths (bp), numbers above the lines indicate intron lengths (bp). (**B**) Exon-intron boundary sequences. Upper-case letters, exonic sequence; lower-case letters, intronic sequence. Bold “**gt**” and “**ag**” highlight that all boundaries except intron 5 follow the canonical GT-AG rule.

The complete genomic sequence of IGF2BP2 gene (GenBank accession PP548073) was obtained by amplifying and assembling products from ZW goose blood DNA using eleven primer pairs (BP2-gF1/R1 to BP2-gF11/R11; Table 1). The locus spans 12,183 bp and consists of 12 exons separated by 11 introns (Figure 3A). Exon lengths range from 24 bp (exon 6) to 511 bp (exon 1), while intron sizes vary between 80 bp (intron 10) and 3773 bp (intron 1). All splice junctions strictly adhere to the canonical GT-AG rule (Figure 3B), confirming accurate mRNA splicing.

The amplicons generated using the same eleven primer pairs (BP2-gF1/gR1 to BP2-gF11/gR11; Table 1) were aligned using SeqMan software (DNASTAR, Madison, USA). Within the approximately 12,183 bp region analyzed, a total of 60 genetic variants were identified, consisting of 54 single nucleotide polymorphisms (SNPs) and 6 insertion/deletion variants (Indels). Of these 60 variants, five were situated in the 5′UTR, six in coding regions, 55 in intronic regions, and one in the 3′UTR (Table 2). Of the coding-region variants, three were synonymous polymorphisms: g.2299delG (Gly47Gly) in exon 1 within the RRM domain, g.10695G>A (Pro434Pro) in exon 9, and g.10927G>A (Arg444Arg) in exon 10 within the KH3 domain. Notably, the deletion of a G base at position 2299 (g.2299delG) results in a frameshift mutation. Further sequencing of DNA samples from the ZW goose population revealed that four sites in exon 1—g.2299delG, g.2304A>C, g.2317G>C, and g.2364C>CTTCT—were in complete linkage disequilibrium (Figure S1).

### 3.3. Tissue Expression Profile of IGF2BP2 mRNA in ZW Geese

**Figure 4:**
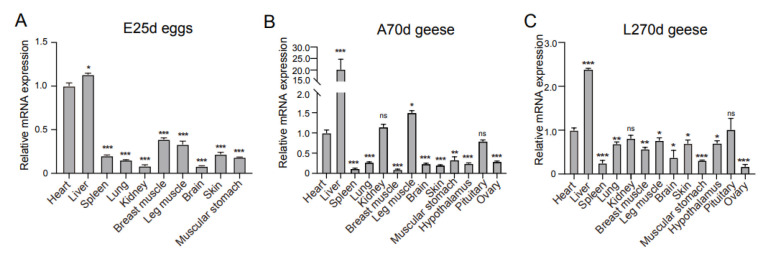
Tissue-specific expression profile of *IGF2BP2* mRNA across three developmental stages in ZW geese. Relative mRNA levels of *IGF2BP2* were analyzed by qPCR in various tissues from female embryonic (E25d) (**A**), growing (A70d) (**B**), and laying geese (L270d) (**C**). Expression was normalized to *β-actin* using the ΔΔCt method (*n* = 4 per tissue). Data are shown as mean ± SEM. Within each panel, asterisks indicate significant differences compared with the tissue showing the lowest expression at that stage (* *p* < 0.05, ** *p* < 0.01, *** *p* < 0.001; ns, not significant).

As shown in Figure 4, qRT-PCR analysis revealed distinct tissue-specific expression patterns of IGF2BP2 mRNA in female ZW geese across three developmental stages: E25d, A70d and L270d. At E25d, IGF2BP2 expression was highest in the muscular stomach—a major site of post-transcriptional regulation during organogenesis—followed by the heart, breast muscle, liver, leg muscle, spleen, lung, brain, and kidney, with the skin showing the lowest level (Figure 4A).

In A70d geese, elevated expression was detected in the liver and leg muscle, consistent with IGF2BP2′s roles in glycogen metabolism and muscle development. Moderate expression occurred in the kidney, heart and pituitary, while lower levels were observed in the muscular stomach, lung, brain, ovary, hypothalamus, and skin. The spleen and breast muscle exhibited the lowest expression (Figure 4B).

At L270d, the liver showed the highest expression, corresponding to its central roles in metabolism and lipid synthesis. The pituitary, heart and kidney displayed relatively high expression, whereas lower levels were found in the leg muscle, lung, skin, breast muscle, brain, muscular stomach, spleen, and ovary (Figure 4C).

### 3.4. Gene Expression Analysis of IGF2BP2-Overexpressed Cells

**Figure 5:**
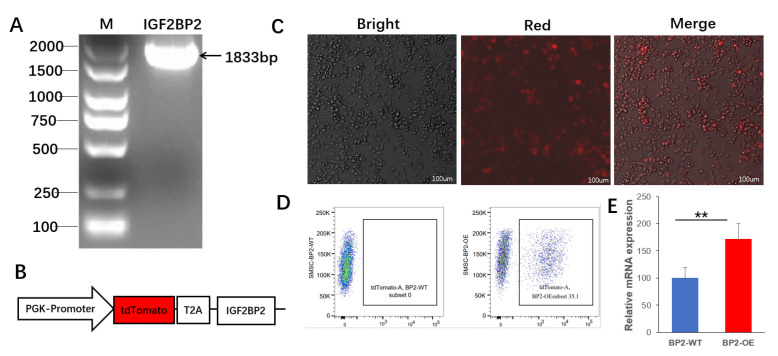
Construction and validation of the *IGF2BP2* over-expression vector in goose SMSCs. (**A**) PCR amplification of the 1833 bp *IGF2BP2* ORF. (**B**) Schematic diagram of the PGK promoter–tdTomato–T2A–IGF2BP2 cassette. (**C**) Bright-field, red-fluorescence and merged images showing robust tdTomato expression after transfection. (**D**) Flow-cytometric enrichment of tdTomato positive cells for subsequent RNA-seq. (**E**) Relative IGF2BP2 mRNA levels in BP2-OE versus BP2-WT groups (mean ± SEM; ** *p* < 0.01).

To investigate the functional role of IGF2BP2 in goose SMSCs, we constructed an overexpression vector (Figure 5A,B) and transfected it into SMSCs (Figure 5C). After three days of culture, successfully transfected cells were isolated by FACS at an efficiency of 35.1% (Figure 5D). The positive cell population exhibited significantly higher expression of IGF2BP2 compared to the wild-type control (BP2-WT; *p* < 0.01; Figure 5E). These results confirm the successful construction and transfection of the BP2-OE vector, supporting its use subsequent functional assays.

**Figure 6:**
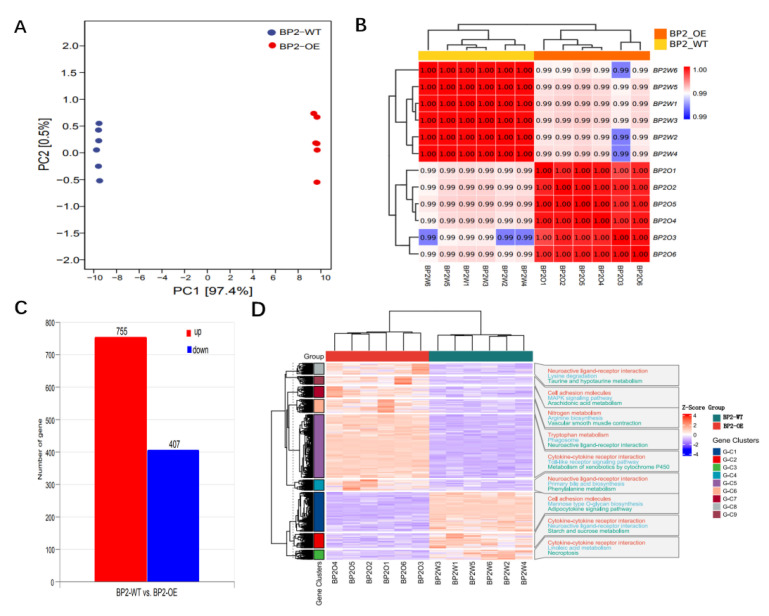
Transcriptome overview of IGF2BP2 over-expression in goose SMSCs. (**A**) Principal component analysis (PCA) of transcriptome profiles from BP2-OE and BP2-WT groups (*n* = 6 per group). Red symbols: BP2-OE (overexpression) replicates; Blue symbols: BP2-WT (control) replicates. (**B**) Correlation analysis of sequencing samples. (**C**) Summary bar chart of DEGs between BP2-OE and BP2-WT groups (*Padj* < 0.05). Red bar: number of up-regulated genes in BP2-OE vs. WT; Blue bar: number of down-regulated genes in BP2-OE vs. WT. (**D**) Z-score heatmap of DEGs and their associated enriched terms.

A total of 12 samples—six from the IGF2BP2-overexpressing group (BP2-OE) and six from the wild-type control group (BP2-WT)—were subjected to transcriptome sequencing. Principal component analysis (PCA) revealed clear separation between the two groups, indicating substantial transcriptomic differences (Figure 6A,B). Differential expression analysis identified 1162 significantly dysregulated genes (*Padj* < 0.05), with 755 upregulated and 407 downregulated in BP2-OE compared to BP2-WT (Figure 6C).

Clustering analysis showed elevated Z-scores in both BP2-WT and BP2-OE groups, whereas gene clusters G-C1 to G-C9 exhibited lower Z-scores, indicating a clear separation in expression profiles between experimental groups and individual gene clusters (Figure 6D). Enrichment analysis revealed that multiple clusters were significantly associated with pathways such as neuroactive ligand-receptor interaction, cytokine-cytokine receptor signaling, and various metabolic processes. Notably, BP2-WT and BP2-OE samples displayed distinct pathway-activation signatures—for example, nitrogen metabolism in BP2-OE and vascular smooth muscle contraction in BP2-WT (Figure 6D). A complete list of DEGs is provided in Table S1, and all raw sequencing data have been deposited in the SRA database under accession PRJNA1369723.

### 3.5. Identification of Key Genes Associated with SMSCs Development

**Figure 7:**
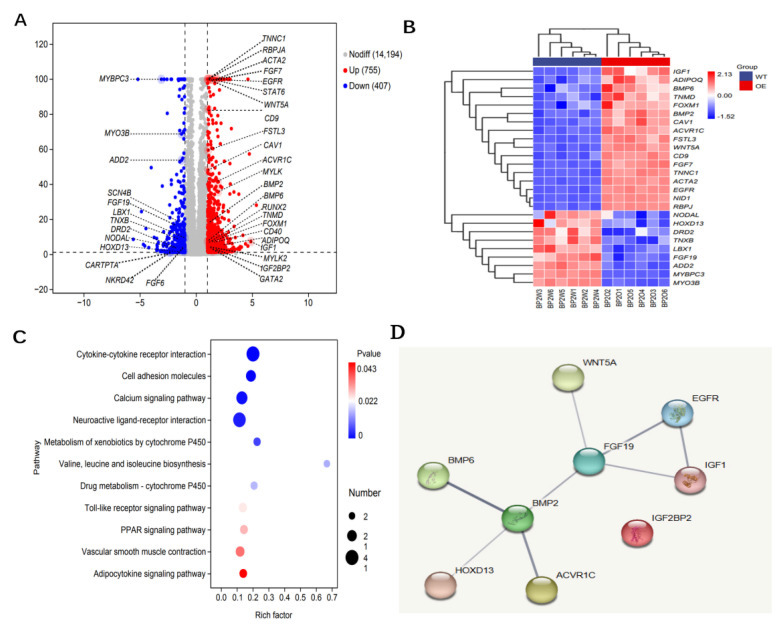
DEG analysis of IGF2BP2-overexpressing cells. (**A**) Volcano plot of transcriptome data; red and blue dots indicate significantly up- and down-regulated genes (|log_2_FC| ≥ 1, FDR ≤ 0.05). (**B**) Heatmap of muscle-development-related DEGs across biological replicates; color scale denotes row-wise Z-scores. (**C**) KEGG pathways enrichment analysis of DEGs. (**D**) Protein–protein interaction network of core muscle-development DEGs.

As an RNA-binding protein, IGF2BP2 plays a critical role in cell growth, metabolism, and development, partly through the stabilization of target mRNAs. Our findings indicate that overexpression of IGF2BP2 in goose SMSCs is associated with substantial transcriptomic alterations. As shown in Figure 7A,B, IGF2BP2 upregulation coincided with increased expression of genes, including IGF1 (*Padj* = 1.57 × 10^−7^), EGFR (*Padj* = 1.00 × 10^−100^), FOXM1 (*Padj* = 1.69 × 10^−7^), and BMP2 (*Padj* = 2.97 × 10^−18^) and BMP6 (*Padj* = 9.95 × 10^−8^). This expression profile is consistent with a phenotype resembling activated progenitor cells or myofibroblasts. Furer transcriptional changes were characterized by the upregulation genes involved in non-canonical Wnt signaling (WNT5A, *Padj* = 1.00 × 10^−100^) and TGF-β signaling pathway (ACVR1C, *Padj* = 7.01 × 10^−41^), alongside the downregulation of key developmental regulators such as FGF19 (*Padj* = 1.82 × 10^−16^), NODAL (*Padj* = 0.0005), and HOXD13 (*Padj* = 0.0104).

Consistent with these findings, KEGG pathway analysis revealed significant enrichment of pathways related to muscle–extracellular matrix communication and metabolic remodeling. The most highly enriched modules were cytokine–cytokine receptor interaction (*p* = 0.043), cell adhesion molecules (*p* = 0.022), neuroactive ligand–receptor interaction, calcium signaling, and Toll-like receptor signaling. Lipid metabolism-related pathways—including PPAR signaling, adipocytokine signaling and valine/leucine/isoleucine biosynthesis—were also enriched. Furthermore, vascular smooth muscle contraction and xenobiotic metabolism by cytochrome P450 showed high enrichment factors, indicating a shift toward oxidative and membrane reorganization processes.

As illustrated in Figure 7D, PPI network analysis clustered core developmental and growth signaling factors—WNT5A, EGFR, BMP6, IGF1 and FGF19—together with HOXD13 and ACVR1C. These results suggest that IGF2BP2 may cooperatively modulate this developmental/metabolic pathway by stabilizing the corresponding mRNAs.

### 3.6. Validation of DEGs by qPCR

**Figure 8:**
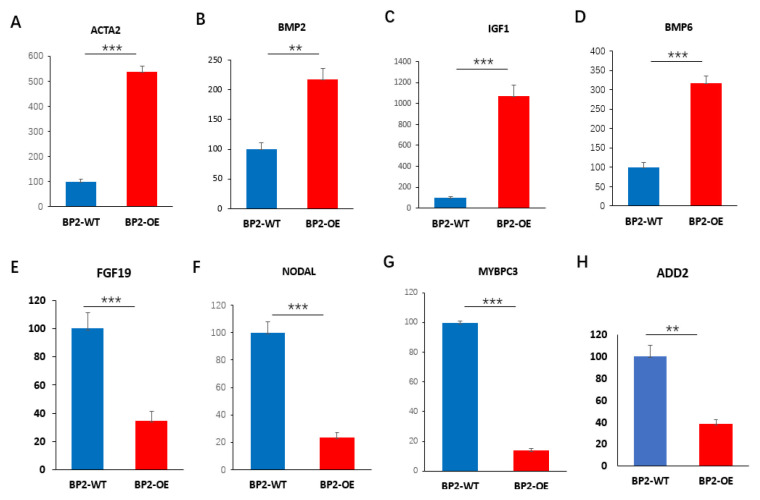
Relative quantitative values for eight selected DEGs obtained from the RNA-seq data. (**A**–**H**) Relative mRNA expression levels of ACTA2, BMP2, IGF1, BMP6, FGF19, NODAL, MYBPC3, and ADD2 genes in BP2-WT and BP2-OE, respectively. ** *p* < 0.01, *** *p* < 0.001; *n* = 6.

To confirm the reliability of the RNA-seq data, we selected eight DEGs for qPCR validation. The qPCR results of these eight DEGs were highly consistent with the RNA-seq data, showing a strong and statistically significant correlation (*p* < 0.01, Figure 8).

## 4. Discussion

IGF2BP2, a central member of the IGF2BP family, contains two RRMs in its N-terminal region and four KH domains in its C-terminal region [23,24,25]. These domains serve as RNA-binding modules in various proteins and are also i in protein–protein interactions, including dimerization [26]. Alternative splicing allows a single gene to generate multiple isoforms with potentially distinct functions [27]. In chickens, three alternative splicing variants of *IGF2BP2* mRNA (cIGF2BP2-X1∼X3) are annotated in the GenBank database. In humans, IGF2BP2 undergoes alternative splicing that can exclude exon 10, which encodes a 43-amino acid region between the KH2 and KH3 domains; a novel isoform lacking a conserved RRM has also been identified in humans and rodents [28]. In this study, we used 5′-RACE to further analyze IGF2BP2 transcripts in ZW geese and identified a cDNA variant missing an N-terminal RRM. Additionally, the IGF2BP2 protein exhibits length variation across species, with amino acid counts as follows: human (599 aa), mouse (592 aa), pig (556 aa), duck (604 aa), chicken (611 aa), pigeon (613 aa) and goose (553 aa). The sequence information obtained here will support future functional study of the goose *IGF2BP2* gene.

The genomic architecture of IGF2BP2 also exhibits considerable variation across species. In mice, cattle and pigeons, the gene consists of 16 exons and 15 introns, whereas humans and pigs possess 15 exons and 14 introns. In contrast, chickens and ducks possess 17 exons and 16 introns. In geese, the IGF2BP2 gene comprises 12 exons and 11 introns, with additional variations in the lengths of individual exons and introns.

Assessing genetic polymorphisms is crucial in livestock breeding for characterizing genotypes and their associations with production, reproduction, and economically important traits [29]. IGF2BP2 has previously been identified as a candidate gene linked to litter size in goats, as well as to growth and reproductive traits in pigeons [9,13]. In this study, we identified 60 variants within the IGF2BP2 genomic sequence in a ZW goose population. Among these were four complete linked sites (g.2299delG, g.2304A>C, g.2317G>C, and g.2364C>CTTCT) located within the RRM domain. The g.2299delG site and its associated haplotype warrant further investigation for their potential influence on economically important traits in geese.

In poultry, SMSCs are a crucial class of stem cells that play a vital role in postnatal skeletal muscle growth, regeneration, and maintenance [30]. Previous studies have established that skeletal muscle development is regulated by a variety of genes and non-coding RNAs [31,32,33,34,35]. A recent study also showed that CTRP3 can modulate the expression of genes involved in skeletal muscle differentiation in geese [36]. In the present study, transcriptomic analysis revealed that overexpression of goose IGF2BP2 significantly altered the expression of 1162 genes. The observed expression pattern indicates that IGF2BP2 overexpression is associated with a transcriptional network promotes proliferation and differentiation. The network is characterized by the upregulation of key mitogenic signals (e.g., IGF1, EGFR), cell cycle regulators (e.g., FOXM1), and BMP pathway components (e.g., BMP2, BMP6), along with the downregulation of developmental regulators such as NODAL and HOXD13.

This transcriptomic reprogramming was further supported by pathway enrichment analysis, which highlighted significant involvement of pathways related to cell adhesion and extracellular matrix organization. The PPI clustering suggests that IGF2BP2 may cooperatively regulate this integrated developmental and metabolic pathway, likely through stabilization of the corresponding mRNAs. Meanwhile, the strong concordance between RNA-seq and qPCR data validates the observed expression changes. Future studies should include experiments such as RIP-qPCR or CLIP to investigate whether IGF2BP2 directly binds to target mRNAs. Such investigations will be essential for clarifying the precise molecular mechanisms through which IGF2BP2 influences the development of goose SMSCs.

## 5. Conclusions

In summary, this study provides a comprehensive characterization of the IGF2BP2 gene in geese, delineating its genomic architecture, spatiotemporal expression patterns, and conserved functional domains. Potential regulatory variants within this gene were also identified. Functionally, we demonstrated that overexpression of goose IGF2BP2 in SMSCs correlated with a transcriptomic profile indicative of enhanced proliferative capacity and altered cell adhesion. Collectively, these findings suggests that IGF2BP2 may serve as a pivotal regulator involved in myogenic processes in geese.

## Figures and Tables

**Table 1:** Primers used in this study.

Primer Purpose	Primer Name	Primer Sequence (5′→3′)	Product Size (bp)
Sex test	CHD1-F CHD1-R	TGCAGAAGCAATATTACAAGT AATTCATTATCATCTGGTGG	466/326
RT-PCR	BP2-F1 BP2-R1	TGCCTGAAGACTTGTTGCATCC CAAGTTGACAACCTCCTGCTCC	1246
	BP2-F2 BP2-R2	GCCACCAGCTTGAGAGCTATTCC GGTGCTCTTTGGTTTGCTCCCTG	1483
5′-RACE	GSP-5R NGSP-5R	GAGGAGGGGAGGGTGAGGAGGTCGG GAGAAGAGAGAAGACAAAGTCCCCC	2292
Genomic DNA cloning and genetic variants identification	BP2-gF1 BP2-gR1	CCCAAAAGCACCCCACCGCACC GCAAATGACACAGCCAAGAGCC	1480
	BP2-gF2 BP2-gR2	CATTGCCCTCTCCATGGACCTT CTCCCTCTCGCATCCCCTAACC	707
	BP2-gF3 BP2-gR3	AGAGGGCATTTGGGTTTGTCAGT CAAATCCCAAGGGCAAAGAGTGT	1245
	BP2-gF4 BP2-gR4	ACACTCTTTGCCCTTGGGATTT AGCCGAGGAAGAGGATGGTTGA	1358
	BP2-gF5 BP2-gR5	ATCCTCTTCCTCGGCTGTCCTC AGGGAGAGGGGGAGCAGCAGGG	836
	BP2-gF6 BP2-gR6	TTCCCTCCTCGGTGCTTAGATG CGGGCTCCTTTCAACATCTGCT	1163
	BP2-gF7 BP2-gR7	TGCCCCGTCGTGGTGTTGGATG TGTCTTCTCCACTTGGCTGCTC	1643
	BP2-gF8 BP2-gR8	CGGCCACCCCTTACCACCCATT CTGTTTGGGGTGATGTTTTGGG	1602
	BP2-gF9 BP2-gR9	ACCATGGTTTAGCACGTCGGAG TTAGGAATGCCACCCCAAACAG	848
	BP2-gF10 BP2-gR10	AGTTCTCGCCAAATCTTCTCCA AGGCAGCCATCAGGAAAAGGGT	1568
	BP2-gF11 BP2-gR11	GTTGTGAATATTCCCGCCTGGT AACCCTCTGCCTATGTTAGTC	923
qPCR analysis	ACTA2-F ACTA2-R	GGTGTGATGGTTGGTATGGGT TTGTAGAAAGAATGGTGCCAG	152
	BMP2-F BMP2-R	CCAACACCGTGCGCAGCTTCC GTGATGGTAGCTGCTGTTGTT	191
	IGF1-F IGF1-R	CTGTGTGGTGCTGAGCTGGTT AGTACATCTCCAGCCTCCTCA	169
	BMP6-F BMP6-R	GGAATTCACGCCTCACCAGCA GGATGTTCATGCAGCACTTGG	174
	FGF19-F FGF19-R	GCACGGGCAGCTCAGGTATTC TGACTCCACTGGCACCGTGTT	202
	NODAL-F NODAL-R	GCAACGTCACCCTGGACATCT CTGAGCGTGCCGGTGAGGTTG	148
	MYPBC3-F MYPBC3-R	GATGTGAGGTGTCTACTAAGG GCACTAAAATTCAACTCTCCA	196
	ADD2-F ADD2-R	CATCGCCTGCTCGACCTCTAC GCTGGCTGCTGTCACCTCGCT	132
	β-actin-F β-actin-R	TCCGTGACATCAAGGAGAAG CATGATGGAGTTGAAGGTGG	224

**Table 2:** SNPs and InDels variations detected in *IGF2BP2* gene.

No	Variations	Region	No	Variations	Region
1	g.240A>G	5′UTR	31	g.8193C>T	Intron 5
2	g.531T>C	5′UTR	32	g.8301G>A	Intron 5
3	g.1597C>A	5′UTR	33	g.9015–9018del	Intron 6
4	g.1973T>C	5′UTR	34	g.9105G>A	Intron 6
5	g.2067–2068AG/CA	5′UTR	35	g.9226T>C	Intron 6
6	g.2124–2137del	5′UTR	36	g.9769T>A	Intron 6
7	g.2299delG	Exon 1	37	g.10383C>T	Intron 8
8	g.2304A>C	Exon 1	38	g.10414G>A	Intron 8
9	g.2317G>C	Exon 1	39	g.10493G>A	Intron 8
10	g.2364C>CTTCT	Exon 1	40	g.10624C>T	Intron 8
11	g.3147T>G	Intron 1	41	g.10635C>T	Intron 8
12	g.4281A>G	Intron 1	42	g.10695G>A	Exon 9
13	g.4487T>C	Intron 1	43	g.10731–10766del	Intron 9
14	g.4489G>A	Intron 1	44	g.10780–10786AATGGCA>CTGATGGC	Intron 9
15	g.4576G>A	Intron 1	45	g.10818C>T	Intron 9
16	g.4600G>C	Intron 1	46	g.10820G>A	Intron 9
17	g.4605G>A	Intron 1	47	g.10824C>T	Intron 9
18	g.4621A>G	Intron 1	48	g.10848T>C	Intron 9
19	g.5102G>A	Intron 1	49	g.10861T>C	Intron 9
20	g.6109T>C	Intron 1	50	g.10869A>G	Intron 9
21	g.6980C>T	Intron 3	51	g.10875–10876TT>CC	Intron 9
22	g.7824G>A	Intron 5	52	g.10927G>A	Exon 10
23	g.7898G>A	Intron 5	53	g.11292T>C	Intron 11
24	g.7951A>G	Intron 5	54	g.11301T>C	Intron 11
25	g.8005A>G	Intron 5	55	g.11318C>T	Intron 11
26	g.8116A>G	Intron 5	56	g.11067C>T	Intron 11
27	g.8121delC	Intron 5	57	g.11868T>G	Intron 11
28	g.8127T>C	Intron 5	58	g.11705C>A	Intron 11
29	g.8138T>C	Intron 5	59	g.11723C>A	Intron 9
30	g.8154G>A	Intron 5	60	g.12082G>C	3′UTR

## Data Availability

The datasets utilized in this study are publicly accessible through online repositories. Specifically, the transcriptome data are deposited in the NCBI BioProject database under accession number PRJNA1369723 (https://www.ncbi.nlm.nih.gov/bioproject/PRJNA1369723, accessed on 27 November 2025).

## Supplementary Materials

The following supporting information can be downloaded at https://www.mdpi.com/article/10.3390/ani16010058/s1, Figure S1: 4 complete linkage loci located in exon 1 region of gIGF2BP2 gene; Table S1: A complete list of DEGs.
