# Supplementary figures and images for: Molecular Characterization and Functional Insights into Goose IGF2BP2 During Skeletal Muscle Development
Source: Animals (Basel). 2025 Dec 24;16(1):58. doi: 10.3390/ani16010058 (PMC12785058; doi:10.3390/ani16010058)

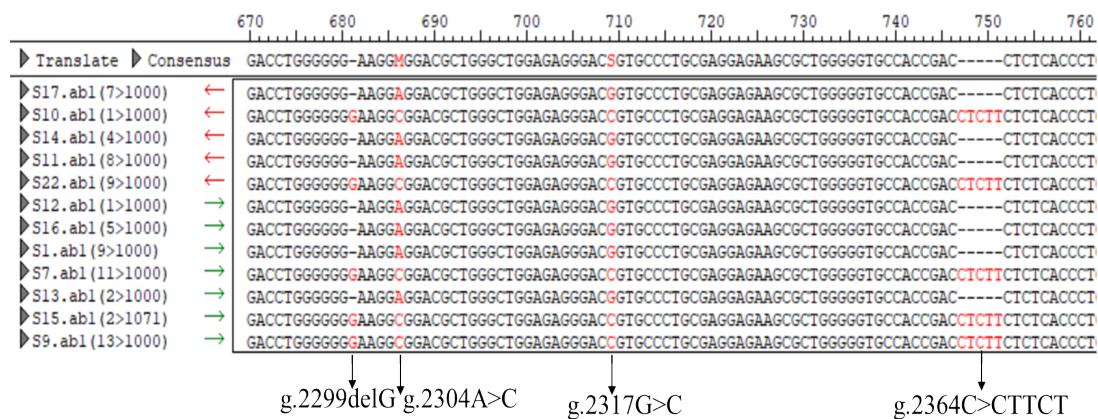

Supplement: Supplementary file 1 [file animals-16-00058-s001.zip › Figure S1.pdf]
